# *Candidozyma auris* Spondylodiscitis: A Case Report from Saudi Arabia and Literature Review

**DOI:** 10.3390/pathogens14090903

**Published:** 2025-09-08

**Authors:** Rakan Sambas, Khalid Bin Aziz, Faisal N. Alqahtani, Hussam Alhathlol, Marwan Alhumaidi, Abdulrahman Alsaedy, Thamer S. Alhowaish

**Affiliations:** 1 Department of Medicine, King Abdulaziz Medical City, Ministry of the National Guard Health Affairs (MNGHA), Riyadh 11481, Saudi Arabia; 2 College of Medicine, King Saud Bin Abdulaziz University for Health Sciences, Riyadh 11481, Saudi Arabia; 3 King Abdullah International Medical Research Center, Riyadh 11481, Saudi Arabia; 4 Department of Neurology, King Abdulaziz Medical City, Ministry of the National Guard Health Affairs (MNGHA), Riyadh 11481, Saudi Arabia

**Keywords:** *Candidozyma auris*, *Candida auris*, fungal spondylodiscitis, multidrug resistance, case report

## Abstract

*Candidozyma auris* (formerly known as *Candida auris*) is an emerging multidrug-resistant fungal pathogen that has become increasingly implicated in healthcare-associated infections; however, its involvement in spondylodiscitis is exceedingly rare. We report the case of a 65-year-old Saudi male with multiple comorbidities who presented with altered mental status and was subsequently diagnosed with *Candidozyma auris* spondylodiscitis and bilateral psoas abscesses. Despite broad-spectrum antifungal therapy and multidisciplinary management, the patient’s condition rapidly deteriorated. This case highlights the significant challenges in diagnosing and managing multidrug-resistant *C. auris* infections and underscores the need for early suspicion, effective source control, and novel antifungal strategies in high-risk populations.

## 1. Introduction

Spondylodiscitis, an infection of the intervertebral disk space and adjacent vertebral bodies, is an uncommon but serious condition, particularly in immunocompromised individuals [1]. While bacterial pathogens are most commonly implicated, fungal etiologies remain rare and diagnostically challenging [2]. *Candidozyma auris* (*C. auris*; formerly known as *Candida auris*), a multidrug-resistant fungal pathogen, has emerged globally as a formidable healthcare-associated organism due to its ability to cause invasive infections, persist in the environment, and resist standard antifungal therapies [3,4,5]. The US Centers for Disease Control and Prevention (CDC) has classified *C. auris* as an urgent public health threat. Similarly, the World Health Organization (WHO) has designated *C. auris* as a top priority for research and public health interventions [6]. Fungal spondylodiscitis is a clinical rarity that poses a diagnostic dilemma due to its non-specific symptoms, slow progression, and difficulty in isolating the organism using conventional microbiological methods [7]. Delayed diagnosis may lead to progressive spinal destruction, neurological compromise, and increased mortality. Given the emerging nature of *C. auris* and its potential for antifungal resistance, recognition of atypical presentations is crucial for timely diagnosis and appropriate management.

In this report, we present a rare case of *C. auris* spondylodiscitis in a 65-year-old patient with multiple comorbidities. Through this case and a review of the current literature, we aim to highlight the clinical presentation, diagnostic workup, therapeutic challenges, and outcomes associated with this unusual manifestation of *C. auris* infection.

## 2. Case Description

A 65-year-old Saudi male with a complex medical history including type two diabetes mellitus, hypertension, adrenal insufficiency, end-stage renal disease (ESRD) on intermittent hemodialysis via a left internal jugular permanent catheter, performed three times per week, and a prior admission complicated by a psoas abscess with destructive spinal changes at L4–L5 and L5–S1 presented to the emergency department with a two-day history of altered mental status, generalized fatigue, and reduced oral intake. His surgical history included a right above-knee amputation and left lower limb angioplasty secondary to gangrene. A severe allergy to trimethoprim–sulfamethoxazole was documented.

On admission, the patient was afebrile and hemodynamically stable but exhibited disorientation and decreased alertness. Physical examination revealed reduced breath sounds bilaterally with right-sided fine crepitations. Cardiovascular and abdominal exams were unremarkable, and there was no peripheral edema or signs of deep vein thrombosis.

Laboratory findings included normocytic anemia with hemoglobin 100 g/L (10 g/dL), leukopenia 3.93 × 10^9^/L (3.93 × 10^3^/µL), thrombocytopenia 105 × 10^9^/L (105 × 10^3^/µL), elevated B-type natriuretic peptide 706.5 pmol/L (5974 pg/mL), hyperuricemia 352 µmol/L (5.91 mg/dL), elevated alkaline phosphatase 548 U/L, and hypocalcemia 2.13 mmol/L (8.52 mg/dL). Initial imaging (Computed tomography (CT) brain, CT cerebral angiography, and CT pulmonary angiography) showed no acute abnormalities. He was admitted for suspected sepsis-related encephalopathy with empiric antibiotic coverage consisting of cefepime (1 g IV once daily), vancomycin (1 g IV once daily), liposomal amphotericin B (7 mg/kg IV once daily), and caspofungin (100 mg IV once daily). The decision to initiate dual antifungal therapy at this stage was influenced by the patient’s prior isolation of *C. auris* and the concern for invasive fungal infection given his comorbidities and severity of presentation.

On the second day of hospitalization, the patient’s neurological status acutely deteriorated, with Glasgow Coma Scale (GCS) dropping to 3/15. He was emergently intubated, required vasopressor support, and was transferred to the intensive care unit (ICU). At this point, empiric antimicrobial therapy was broadened further by switching cefepime to meropenem (1 g IV once daily) and adding metronidazole (500 mg IV twice daily), ampicillin (2 g IV twice daily), and acyclovir (220 mg IV once daily), while continuing dual antifungal coverage with liposomal amphotericin B (7 mg/kg IV once daily) and caspofungin (100 mg IV once daily).

In the current admission, the infectious diseases team was consulted and recommended spinal magnetic resonance imaging (MRI) to evaluate for deep-seated infection, given the history of destructive changes at L4–L5 and L5–S1 with an associated psoas abscess during the prior admission four months earlier. At the prior admission, the abscess had been empirically treated with meropenem (500 mg IV daily), vancomycin (1 g IV every 48 h), and amikacin (200 mg IV daily) for three weeks, after which the patient was discharged in stable condition. A culture was taken, and the initial potassium hydroxide (KOH) preparation of the abscess specimen showed no fungal elements. However, the culture later showed scanty growth of *C. auris* after 13 days of incubation, which was identified by matrix-assisted laser desorption/ionization time-of-flight (MALDI-TOF) mass spectrometry. The culture was maintained for four weeks, during which no additional fungal organisms were isolated. These results only became available to the treating team during the current hospitalization. No antifungal susceptibility testing was performed for this initial isolate. The culture finalized after the patient had already been discharged, and by the time antifungal susceptibility was requested, the culture had already been discarded. 

**Figure 1:**
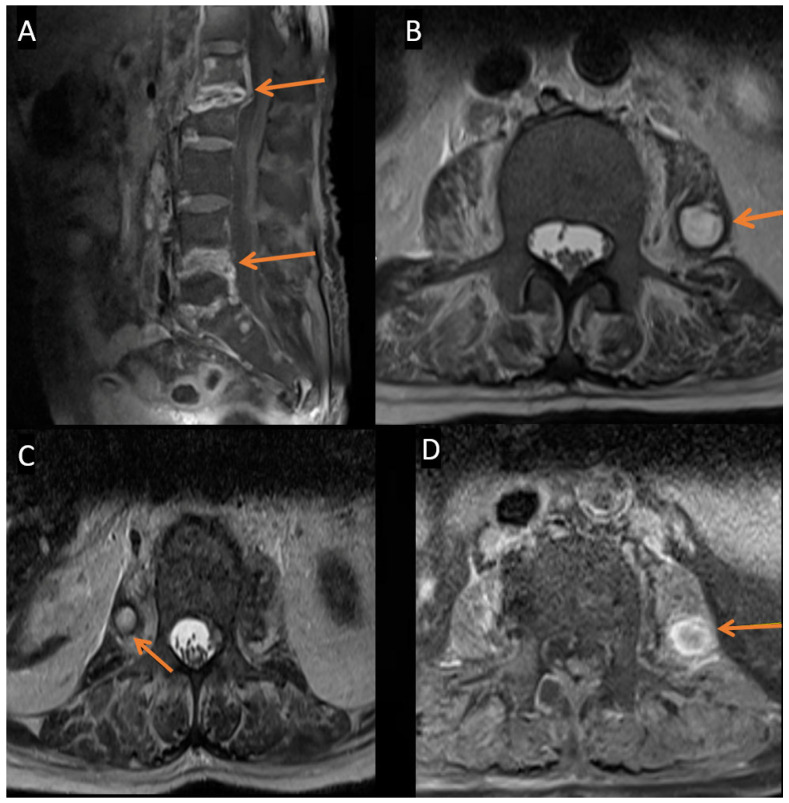
(**A**) Sagittal T1-weighted post-contrast MRI demonstrates enhancing destructive changes involving the L1–L2, L4–L5, and, to a lesser extent, L5–S1 vertebral endplates and intervertebral disks, associated with enhancing epidural soft tissue extension (arrows). These findings are consistent with multilevel spondylodiscitis and epidural involvement. (**B**–**D**) Axial T2-weighted and T1-weighted post-contrast images show well-defined T2 hyperintense lesions within the bilateral psoas major muscles, exhibiting marked peripheral enhancement (arrows), consistent with intramuscular abscesses.

**Figure 2:**
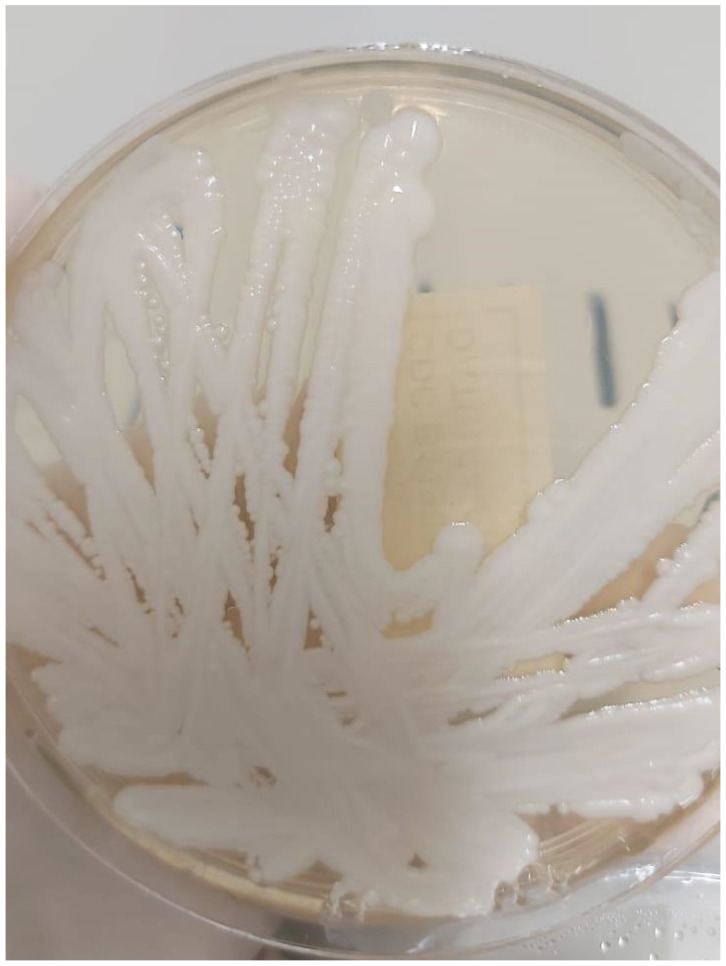
Sabouraud dextrose agar demonstrating robust growth, showing creamy-white and smooth colonies of C. auris. This isolate was confirmed to be susceptible to amphotericin B, micafungin, and anidulafungin, but resistant to fluconazole. For further description, refer to Appendix A.

During the current admission, spinal MRI again revealed multilevel spondylodiscitis at L4–L5 and, to a lesser extent, L5–S1, along with bilateral psoas abscesses (Figure 1). Extensive workup of the previous abscess, including *Tuberculosis* (TB) polymerase chain reaction (PCR), acid-fast bacilli staining, tissue cultures, surgical pathology, and *Brucella* serology, was negative. A repeat culture obtained during the current admission, showed no fungal elements on the initial potassium hydroxide (KOH) preparation. However, fungal culture turned positive after nine days of incubation, yielding heavy growth of *C. auris*, which was identified by MALDI-TOF (Figure 2). The culture was maintained for four weeks, during which no additional fungal organisms were isolated. For this second isolate, Etest susceptibility testing was performed and interpreted according to the CDC’s tentative breakpoints, demonstrating susceptibility to amphotericin B, micafungin, and anidulafungin, but resistance to fluconazole [8]. The corresponding minimum inhibitory concentrations (MICs) are presented in Table 1. These findings confirmed the diagnosis of *C. auris* spondylodiscitis with associated psoas abscesses.

Despite medical therapy, source control was not feasible; interventional radiology, general surgery, and spine surgery all deemed the abscesses too small for drainage and the patient too frail for surgical intervention. After a period of transient stabilization and transfer to the medical ward, the patient suffered a cardiac arrest on day four post-ICU discharge. Resuscitation efforts were unsuccessful, and the patient was pronounced deceased and was laid to rest with no formal autopsy.

## 3. Discussion

*Candidozyma auris* spondylodiscitis is an exceedingly rare but emerging clinical entity, posing significant diagnostic and therapeutic challenges. The present case from Saudi Arabia represents, to our knowledge, the first reported instance in the country, highlighting the expanding geographic footprint of this multidrug-resistant yeast. *C. auris* infections of the spine remain uncommon where fungal spondylodiscitis overall accounts for only a small fraction of vertebral infections [9]. However, *C. auris’s* global emergence as a hospital-associated pathogen demands heightened clinical vigilance even for atypical presentations. Our patient’s extensive comorbidities (diabetes, renal failure, hypertension, and adrenal insufficiency) and recent hospital exposures likely predisposed him to an invasive *C. auris* infection, aligning with risk factors noted in prior reports [10,11,12].

**Figure 3:**
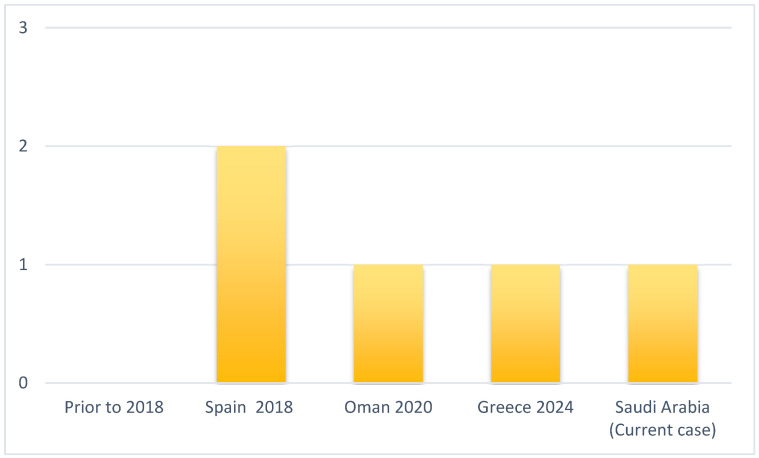
This figure illustrates the emergence of *Candidozyma auris* in recent years, with a rising number of reported cases.

As Figure 3 shows, *C. auris* spondylodiscitis has only been reported in the literature recently, reflecting the recent emergence of this multidrug-resistant yeast and improvements in its detection and recognition. The global incidence of *C. auris* infections has risen sharply in recent years. For example, in the United States, reported *C. auris* cases jumped from 329 in 2018 to over 1000 by 2021, which has expanded opportunities for rare manifestations such as vertebral infection [13]. Concurrently, advances in laboratory diagnostics (e.g., MALDI-TOF mass spectrometry) now enable accurate identification of *C. auris*, whereas earlier cases may have been misidentified as other *Candida* species [14]. Heightened clinical awareness and typical hospital-associated risk factors, such as prolonged ICU stays, invasive devices, or immunosuppression, further contribute to the recognition of *C. auris* spondylodiscitis as an emerging entity.

Three published cases of *C. auris* spondylodiscitis (from Oman, Greece, and Spain) demonstrate notable differences in clinical course, infection route, management, and outcomes [10,11,12]. In Oman, a 50-year-old man with comorbidities developed hospital-acquired *C. auris* spondylodiscitis after multiple surgeries, presenting with back pain and an epidural abscess [10]. He underwent surgical decompression and stabilization, and tissue cultures grew *C. auris*; treatment with caspofungin led to complete resolution of the infection by four months. In Greece, a 73-year-old male developed spondylodiscitis several months after spinal kyphoplasty; this case was attributed to *C. auris*, likely acquired during a brief ICU stay (skin colonization), with the surgical wound serving as the portal of entry [11]. The Greek patient’s infection evolved insidiously where initial biopsies were culture-negative and broad-spectrum antibiotics failed to help, and ultimately required surgical debridement and stabilization; *C. auris* was isolated from wound cultures, and prolonged micafungin therapy (≥9 months) resulted in clinical improvement and normalization of inflammatory markers. By contrast, a Spanish ICU outbreak (2016–2017) involving 41 *C. auris* candidemia cases reported high mortality (30-day mortality ~41%) and several late sequelae [12]. Five patients (~12%) in that outbreak developed severe metastatic complications (e.g., endocarditis or spondylodiscitis) despite appropriate echinocandin therapy, but only two patients had spondylodiscitis specifically [9]. These Spanish cases exemplify hematogenous spread to the spine from *C. auris* candidemia, in contrast to the likely direct inoculation or contiguous wound spread seen in the Oman and Greece cases. Together, these reports show that *C. auris* spondylodiscitis can arise via different pathways and require aggressive management (combining surgery with antifungals), with outcomes ranging from full recovery to serious complications in critically ill patients [10,11,12].

Across these cases, in Oman, Greece, Spain, and now Saudi Arabia, common clinical features emerge. Patients were typically older, often with recent surgeries or ICU admissions, reflecting how *C. auris* primarily exploits healthcare-associated risk factors. Clinical presentation was insidious in all cases, with back pain being the chief complaint; fever and systemic signs were mild or absent, which can delay suspicion of spondylodiscitis. Diagnostic confirmation hinged on invasive sampling (spinal biopsy or surgical specimens) and advanced mycological identification. Conventional lab methods may misidentify *C. auris* as other yeasts; indeed, definitive identification in reported cases was achieved via MALDI-TOF mass spectrometry or DNA sequencing [7,8]. Such technology is crucial, as early species recognition guides appropriate antifungal choice and infection control measures. All reported patients received systemic antifungal therapy with an echinocandin (caspofungin or micafungin), consistent with current recommendations for *C. auris*, which is often resistant to fluconazole and other azoles [9]. In two cases (Oman and Greece), surgical intervention (debridement and stabilization) was performed prior to antifungal therapy, which likely contributed to source control and favorable outcomes [10,11]. By contrast, the Spanish outbreak patients, many of whom were poor surgical candidates, highlight that medical therapy alone may be insufficient in severe disseminated infection, as evidenced by the high complication and mortality rates [12] (Table 2).

For clinicians, our case and the comparative literature offer several key lessons. First, clinicians should maintain a high index of suspicion for fungal etiologies like *C. auris* in cases of culture-negative spondylodiscitis that do not respond to conventional antibacterial therapy, especially in patients with relevant risk factors (ICU stay, prior candidemia, or known *C. auris* colonization during hospital outbreaks). Early consultation with infectious disease specialists is advisable to guide appropriate diagnostic testing (e.g., tissue biopsy for culture and molecular identification) and to implement prompt antifungal therapy. Second, infection control precautions are paramount upon identifying *C. auris*: patients should be isolated and hospital environments rigorously disinfected, given the organism’s ability to persist and spread [15]. Third, management of *C. auris* spondylodiscitis likely requires a multimodal approach, as illustrated by the reviewed cases, combining surgical debridement (when feasible) with prolonged antifungal therapy. Echinocandins are the first-line treatment due to *C. auris’s* frequent multidrug resistance, but therapy should be tailored to susceptibility results if available [16,17,18,19]. Finally, clinicians must be aware that treatment courses need to be lengthy (often at least 6–8 weeks) and that close clinical and radiologic follow-up is necessary to ensure resolution of infection [13,19]. The present case reinforces these points. Despite the rarity of *C. auris* in the spine, recognizing its possibility led to timely targeted therapy.

## 4. Conclusions

To conclude, *C. auris* spondylodiscitis is an emerging infectious disease challenge with only a few cases reported worldwide. Our Saudi Arabian case adds to the growing literature and highlights the importance of considering this pathogen in the differential diagnosis of vertebral infections, particularly in healthcare-associated contexts. Comparing our experience with cases from Oman, Greece, and Spain, we find that while clinical presentations and management principles are similar to other *Candida* spinal infections, the multidrug-resistant and outbreak-prone nature of *C. auris* raises the stakes considerably. Ongoing surveillance, rapid diagnostic capabilities, and adherence to stringent infection control and treatment protocols will be crucial as we confront this “superbug” in musculoskeletal infections. Further research is needed to establish evidence-based guidelines for managing *C. auris* spondylodiscitis, including optimal antifungal regimens and the role of adjunctive surgical therapy. Clinicians should remain vigilant for this rare manifestation and report new cases, as each adds valuable insight into the behavior and best management of *C. auris* in the spine.

## Figures and Tables

**Table 1:** Antifungal susceptibility results of *Candidozyma auris* isolate with CDC tentative MIC breakpoints.

Antifungal Agent	CDC Tentative MIC Breakpoint (µg/mL)	Current Isolate MIC (µg/mL)	Interpretation
Fluconazole	≥32 = Resistant	≥256	Resistant
Voriconazole *	No established breakpoint	2	Resistant
Caspofungin	≥2 = Resistant	NA	NA
Amphotericin B	≥2 = Resistant	0.5	Susceptible
Anidulofungin	≥4 = Resistant	≤0.125	Susceptible
Micafungin	≥4 = Resistant	≤0.125	Susceptible

Abbreviations: MIC, minimum inhibitory concentration; CDC, Centers for Disease Control and Prevention; NA, not available. * No CDC breakpoints are established for voriconazole. The isolate’s high MIC was interpreted as resistant based on expert microbiology review and the known cross-resistance between voriconazole and fluconazole in *Candidozyma auris*.

**Table 2:** Published cases of *Candidozyma auris* spondylodiscitis, including the present case.

Study Name	Patient (Age/Sex, Comorbidities)	Presenting Signs and Symptoms	Spinal Level	Medical Therapy	Dose	Surgical Therapy	Length of Treatment, Months	Follow-Up, Months
Present case (Saudi Arabia)	M/65; T2DM, HTN, adrenal insufficiency, ESRD on dialysis; prior amputation and angioplasty	Reduced level of consciousness, fatigue, and poor oral intake	L4–L5, L5–S1	Caspofungin	100 mg IV once daily	None	1.32	None (patient died)
				Amphotericin B	7 mg/kg IV once daily			
Supreeth et al., 2020 (Oman) [10]	M/50; SCD, DM, multiple surgeries	Progressive low back pain radiating to bilateral lower limbs and intact neurology	L4-L5	Caspofungin	70 mg IV load then 50 mg daily	Debridement, posterior decompression and stabilization	2	6
Langourani-Kosteletou et al., 2024 (Greece) [11]	M/73; DM, prior MI, pacemaker; ICU stay; post-kyphoplasty	Back pain and tenderness, low-grade fever, and intact neurology	T12-L1	Micafungin	100 mg IV daily	Debridement, posterior decompression and stabilization	9	7
Ruiz-Gaitán et al., 2018 (Spain) [12]	M/66, abdominal surgery	NA	NA	Anidulafungin, Liposomal Amphotericin B then Anidulafungin, Posaconazole (Pt 1)	NA	None	6 (Pt 1)	NA
	M/42, polytrauma			Anidulafungin then Posaconazole (Pt 2)			9 (Pt 2)	

Abbreviations: T2DM, type 2 diabetes mellitus; HTN, hypertension; ESRD, end-stage renal disease; SCD, sickle cell disease; MI, myocardial infarction; ICU, intensive care unit; IV, intravenous; NA, not available.

## Data Availability

The raw data supporting the conclusions of this article will be made available by the authors on request.

## Abbreviations

The following abbreviations are used in this manuscript:

CDC	Centers for Disease Control and Prevention
WHO	World Health Organization
*C. auris*	*Candidozyma auris*
ESRD	End-stage renal disease
Hb	Hemoglobin
BNP	B-type natriuretic peptide
BUN	Blood urea nitrogen
CT	Computed tomography
GCS	Glasgow Coma Scale
IV	Intravenous
TB	Tuberculosis
PCR	Polymerase chain reaction
ICU	Intensive care unit
MALDI-TOF	Matrix-assisted laser desorption/ionization time-of-flight
MIC	Minimum inhibitory concentration
MS	Mass spectrometry (used as part of “Vitek MS”)
FDA	Food and Drug Administration
BD	Becton, Dickinson and Company (referenced in “Bruker FDA-approved MALDI Biotyper (BD)”)
T2DM	Type 2 diabetes mellitus
HTN	Hypertension
SCD	Sickle cell disease
MI	Myocardial infarction
NA	Not available

## Appendix A

### Interpretation of Sabouraud Dextrose Agar

Macroscopic features of *Candidozyma auris* may aid in preliminary identification but are insufficient for definitive differentiation from other *Candidozyma* species. Microscopically, *C. auris* exhibits two main morphologies: some strains form aggregates, while others consist of singular budding yeast cells, measuring approximately 2.0–3.0 × 2.5–5.0 µm, with an ovoid to elongate shape. Similar to *Candidozyma glabrata*, *C. auris* typically does not form pseudohyphae on cornmeal or rice Tween 80 agar, although rudimentary pseudohyphae have been observed under certain growth conditions.

Recent software updates for systems such as Vitek 2 have improved the ability to biochemically distinguish *C. auris* from closely related species like *C. haemulonii* and *C. duobushaemulonii*. Proteomic methods, particularly matrix-assisted laser desorption/ionization time-of-flight (MALDI-TOF) mass spectrometry using the Vitek MS platform and BioMérieux Knowledge Base version 3.2, enable rapid and accurate species-level identification, outperforming traditional phenotypic systems.

Accurate identification can also be achieved using the Bruker Biotyper MALDI-TOF system with either the FDA-approved MALDI Biotyper CA System library (Version Claim 4) or the research-use-only libraries (e.g., Version 2014 [5627]).
